# Paraquat resistance mutations have differential effects on plant fitness in two rice cultivars

**DOI:** 10.1042/BCJ20240683

**Published:** 2025-04-23

**Authors:** Jared B. Fudge, Teresa B. Fitzpatrick

**Affiliations:** Department of Plant Sciences, University of Geneva, Geneva 1211, Switzerland

**Keywords:** herbicide resistance, natural variation, paraquat, polyamines, rice

## Abstract

Paraquat (PQ) is a fast-acting non-selective herbicide widely used globally to eradicate weeds. The emergence of weed resistance has fueled the drive to understand molecular mechanistic aspects and develop crops resistant to the herbicide. The transport of PQ is mediated by members of the L-amino acid transporter family, which are prime targets for the development of resistance. However, these transporters also facilitate the transport of natural essential molecules such as polyamines and thiamine (vitamin B_1_), at least in Arabidopsis, but have not undergone rigorous investigation in crops. Here, we report on the disruption of the polyamine transporter *PUT3* in two *japonica* rice cultivars. Both rice *put3*-mutant alleles are resistant to PQ and display low percentage germination concomitant with altered polyamine profiles, whereas thiamine is unchanged. Notwithstanding, seedlings that germinate behave like wildtype in the Tainung 67 cultivar, whereas further growth and development is strongly impaired by the disruption of *PUT3* in the Hwayoung cultivar. The growth phenotype could be complemented by ectopic expression of *PUT3*, which also restores the polyamine profile thus linking the defects to disruption of the gene. Our study provides biological insight into the divergent characteristics of rice cultivar tissues as a function of their polyamine profile and a warning to exercise caution upon disruption of transporters to facilitate PQ resistance in crops as this may also lead to severe fitness penalties.

## Introduction

Paraquat (PQ, also known as methyl viologen) ranks third globally as the most widely used herbicide [1], e.g., approximately 4500 tons were used in the USA in 2018 (https://water.usgs.gov/nawqa/pnsp/usage/maps/). It is mainly applied to control weeds in agricultural settings, notably in non-tillage practices, as the compound kills plants within hours and is rapidly inactivated upon adsorption to soil colloids, thus permitting the sowing of crops immediately after application [1]. PQ works upon uptake and transport to the chloroplast, wherein it impairs photosynthesis by inhibiting electron transport through photosystem I generating toxic levels of reactive oxygen species (ROS) that severely damage the plant [2]. However, PQ resistance has emerged among weeds due to persistent use [3] and is thereby a potential threat to agriculture. In weeds, resistance mechanisms are related to PQ sequestration in the vacuole or enhanced capacity to quench ROS [2]. Further mitigation of the risk of PQ-resistant weeds is a major goal that can be addressed by studying tolerance or resistance mechanisms to PQ in crops. To date, there is very limited understanding of how crops could be exploited mechanistically to manage susceptibility to PQ [1].

*Arabidopsis thaliana* (Arabidopsis) is the most studied model plant species in terms of PQ resistance mechanisms at the molecular level [1]. Through the use of *Arabidopsis* natural variants and mutational studies, tolerance mechanisms include ecotypes that carry single-nucleotide polymorphisms or are mutated in transporters that lead to impaired uptake at the cellular level, enhanced cellular extrusion or sequestration (in the vacuole), or indirectly through sufficient ROS scavenging [4–11]. For example, *paraquat resistant1* (*par1*) is impaired in subcellular transport of PQ apparently via the Golgi apparatus and displays enhanced resistance to the herbicide [6]. By contrast, the overexpression of *PAR1* leads to enhanced susceptibility to PQ [6]. *PAR1* is a deletion mutant in one of the five-membered *polyamine uptake transporter* (*PUT*) family in *Arabidopsis*, with *PAR1* representing *PUT2*. Another member of the *PUT* family in *Arabidopsis* (*PUT3*) localized to the plasma membrane is also implicated in the transport of PQ, resulting from the identification of a mutant referred to as *resistant to methyl viologen1* (*rmv1*) [5]. The latter study also reported natural accessions of *Arabidopsis* (e.g., Nos-d, Est-1, Sha, Tamm-2, RRS-10) that have enhanced tolerance to PQ. All of these varieties carry a single base substitution at locus At5g05630, which encodes the PUT3 transporter causing an amino acid change from isoleucine 377 to phenylalanine found in the ninth transmembrane domain and is linked to the loss of PQ sensitivity [5].

Whereas PQ is a synthetic agent, the natural substrates of the PUT family are polyamines as the name implies [5,12]. Polyamines are ubiquitous cationic, aliphatic compounds implicated in numerous developmental processes and stress responses in both plants and animals [13,14]. For example, in plants, polyamines are involved in embryo development, seed germination and seedling establishment, vascular development, and play a key role in reproductive processes, as well as senescence [13]. Alteration of polyamine levels has been shown to affect cellular abiotic stress responses to drought, salinity, and temperature responses, as well as biotic plant–microorganism interactions [13]. The mechanisms of action of polyamines in both developmental and stress responses are thought to involve stabilization of macromolecules such as nucleic acids and their implication in translation regulation of specific transcripts, or their catabolism that indirectly leads to ROS production [13]. As multifunctional metabolites, homeostasis of polyamine compounds is essential and is mediated through biosynthesis, degradation, conjugation, and transport [13–15]. While polyamine metabolism has been well studied in several plant species, less is known about transport. Interestingly, abundant plant polyamines such as putrescine, spermidine, and spermine are known to compete and impede PQ uptake, indicating a shared transport route [5,16]. Arabidopsis PUT3, the mutant of which is known as *rmv1* as described above, transports polyamines as well as thiamine (vitamin B_1_) [17]. Thiamine in its pyrophosphorylated form is a coenzyme for key reactions of central metabolism [18]. Its biosynthesis *de novo* is mainly associated with photosynthetic tissues but must be transported to distal (non-photosynthetic) tissues for cell energy supply therein [17]. PUT3 facilitates long-distance transport of thiamine in Arabidopsis [17]. Thus, while PQ tolerance is a desirable trait in crop plants through the disruption of the corresponding transporter, their implication in natural-metabolite transport must also be considered. The *PUT* family in rice has also been studied, which has three homologs referred to as *PUT1-3* [12,19,20]. Their transport properties for polyamines have been studied by heterologous expression in yeast and Arabidopsis, tentatively indicating polyamine transport [12,20], but have not undergone rigorous characterization in rice itself. A transgenic rice line with reduced expression of *PUT2* (homolog to Arabidopsis *PAR1*) induced by RNA interference in the Nipponbare variety shows enhanced tolerance to PQ [6], and the disruption of *PUT1-3* by CRISPR in the Xidao#1 variety also leads to PQ-tolerance [19].

Here, we made use of two available rice T-DNA insertion lines in *PUT3* in the Hwayoung (HY) and Tainung 67 cultivar backgrounds. We studied the impact of the mutations on PQ tolerance, as well as growth and development. In addition, we profiled the polyamine and thiamine concentrations in selected tissues to assess for differential distribution as a function of the *PUT3* transporter. Validation of phenotypes and metabolic profiles was assessed by complementation with the *PUT3* transgene.

## Results

### Loss of *PUT3* in rice confers PQ resistance but development and polyamine levels are impaired

**Figure 1:**
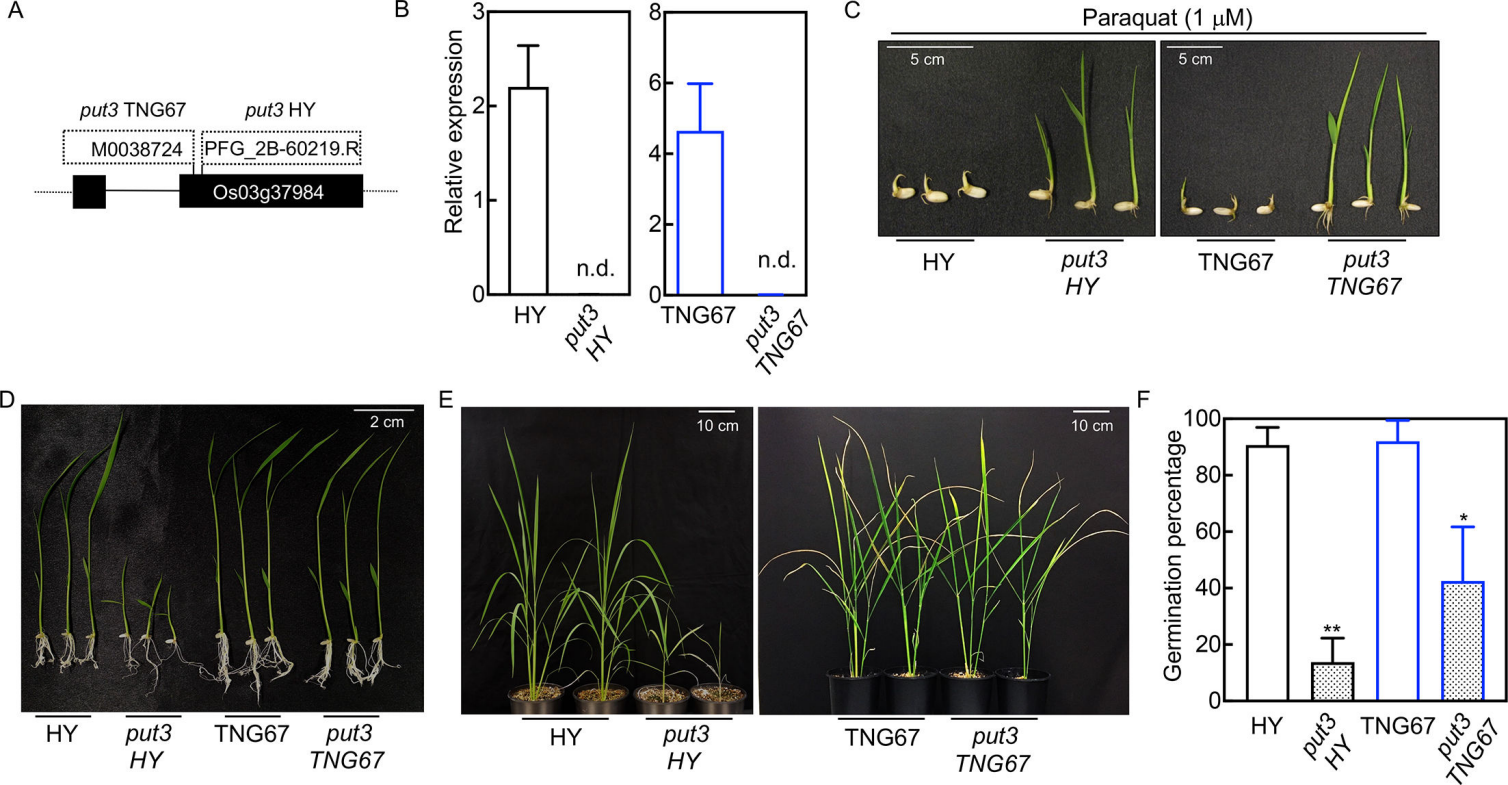
Phenotypic comparison of rice *put3* T-DNA insertion lines from different cultivars. (**A**) Schematic representation of the genomic structure of rice *PUT3:* black boxes, solid, and dashed lines represent exons, intron, and untranslated regions, respectively. T-DNA insertion sites are denoted by the rectangular boxes outlined by dashed lines. (**B**) RT-qPCR of *PUT3* expression in rice *put3* lines compared with the corresponding wild type cultivar. Plants were grown in ½ MS media under 16-hour illumination at 200 μmol photons m^-2^ s^-1^ at 28°C and 8-hour darkness at 20°C. Shoot tissue was harvested at ZT 6 from 12-day-old seedlings. Transcripts were normalized to *ACT1* (Os05g36290). Data are the mean ± SD of *N* = 3 or 4. n.d. implies not detected. (**C**) Phenotypes of rice *put3* lines compared with the corresponding wild type grown in the presence of 1 μM paraquat (PQ). Seeds were grown as in (**B**) supplemented with 1 μM PQ. Plants were photographed 10 days after germination. Scale bars represent 5 cm. (**D**) Seedlings as in (**C**) in the absence of PQ. (**E**) Phenotype of plant lines as indicated grown under greenhouse conditions. Photographs were captured at 71 days after germination for HY and *put3* HY, and 159 days after germination for TNG67 and *put3* TNG67. Scale bars represent 10 cm. (**F**) Percentage germination of seeds as indicated. Data are the mean ± SD of *N* = 4. Asterisks represent statistically significant differences for *P<0.05 and **P<0.01 as determined by a Student’s *t*-test.

In rice, there are three homologs within the *PUT* family, referred to as *PUT1-3* (Os02g0700500/Os02g47210, Os12g0580400/Os12g39080, Os03g0576900/Os03g37984, Japanese, and American annotations, respectively) [12,20]. Here, we focused on the characterization of *PUT3* that is the closest paralog of Arabidopsis *PUT3* (*rmv1*) implicated in PQ and polyamine transport [12,21]. T-DNA insertion mutants were available in the HY cultivar (PFG_2B-60219.R) carrying T-DNA derived from pGA2707 [22] and the Tainung 67 (TNG67) cultivar (M0038724) carrying T-DNA derived from pTag8 [23] (Figure 1A). Transcript levels could not be detected by real-time quantitative PCR (RT-qPCR) in either line and were annotated as *put3* HY and *put3* TNG67, respectively (Figure 1B). Notably, transcript levels of the other *PUT* paralogs (*PUT1*/*2*) were not altered in either *put3* HY or *put3* TNG67 compared with the corresponding wild type (Supplementary Figure S1). Although, we noted that transcript levels of *PUT1* were an order of magnitude lower in shoot tissue of TNG67 compared with HY and lower than *PUT2* in both cultivars (Supplementary Figure S1).

In contrast with wild type cultivars, germinated seeds of both of the rice mutants were tolerant to the presence of PQ in the culture medium, indicating the loss of function of PUT3 (Figure 1C). Under standard growth conditions, we observed that *put3* HY seedlings were developmentally impaired compared with the corresponding wild type (HY) (Figure 1D and E). By contrast, *put3* TNG67 seedlings appeared like the corresponding wild type (TNG67) throughout development (Figure 1D and E). Nonetheless, we observed an impairment in the percentage of seeds germinating in both *put3* HY (20%) and *put3* TNG67 (50%) compared with the respective wild type (Figure 1F).

**Figure 2:**
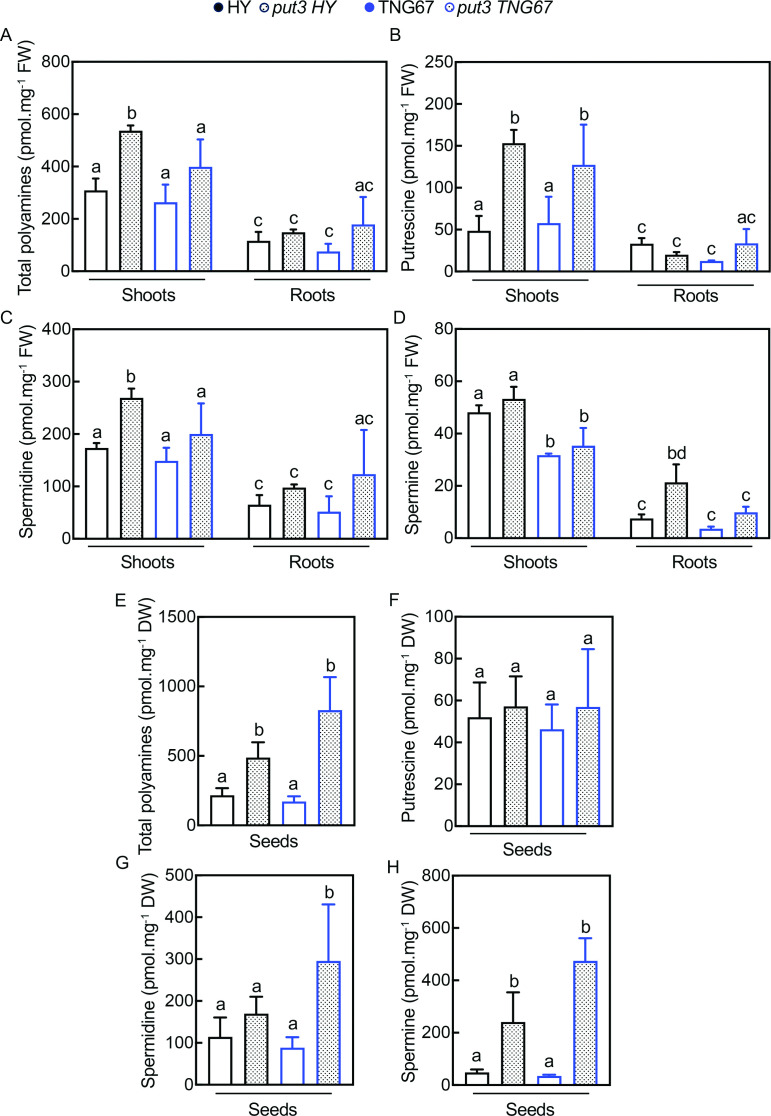
Polyamine profiles of rice *put3* T-DNA insertion lines compared with the wild type cultivar. (**A**) Total polyamine levels in shoots and roots of 10-day-old seedlings grown in ½ MS media under 12-hour illumination at 200 μmol photons m^-2^ s^-1^ at 28°C and 12-hour darkness at 20°C. Tissues were harvested at ZT 6. (**B–D**) Individual polyamine levels for putrescine, spermidine, and spermine in samples as in (**A**). (**E**) Total polyamine levels of mature whole seeds of plants grown under greenhouse conditions. (**F–H**) Individual polyamine levels for putrescine, spermidine, and spermine in samples as in (**E**). Statistically significant differences (*P*<0.05) between means of genotypes within each tissue type were determined by one-way ANOVA with multiple comparisons and Dunnett’s test and are denoted by different letters. Data are the mean ± SD of *N* = 3 or 4.

Next, we investigated polyamine levels in all lines using an established HPLC method that measures the diamine putrescine (Put), the triamine spermidine (Spd), and the tetramine spermine (Spm) [17]. The total level of polyamines measured was significantly increased in shoot tissue of *put3* HY compared with wild type, whereas that of *put3* TNG67 was not significantly different to wild type (Figure 2A). There was no statistically significant difference in total polyamine concentration of root tissues among the genotypes, although levels were generally lower in shoot tissues (Figure 2A). The increase in polyamine concentration of shoot tissue for *put3* HY was proportionally mainly contributed by Put and Spd as the most abundant polyamines in this tissue (Figure 2B and C). We also observed that the Spm concentration (albeit a less-abundant polyamine) was higher in HY shoots compared with TNG67 but not significantly different in the corresponding mutants (Figure 2D). Spm was significantly increased in root tissue of *put3* HY but not that of *put3* TNG67 (Figure 2D). We also measured the total polyamine concentration of mature unpolished rice seeds and observed the enhanced levels for both *put3* HY and *put3* TNG67 (Figure 2E). These increases were proportionally mainly contributed by Spm in this organ and to a lesser extent by Spd, whereas Put was not significantly different among the genotypes (Figure 2F–H). We also measured the vitamin B_1_ concentration by HPLC [24] in shoot and root tissue, as well as unpolished seeds of all genotypes and observed no significant differences between mutant and wild type, although the concentration in shoots was higher than roots and that of seeds of HY was slightly higher than TNG67 (Supplementary Figure S2A and S2B).

Taken together, the loss of *PUT3* confers PQ resistance to the rice cultivars studied here and has previously been observed for the Nipponbare cultivar [6]. Li et al. [6] did not report a morphological phenotype for the rice *put3* knockdown line in the Nipponbare cultivar. Here, we observe major stunting of growth in HY but not TNG67 and reduced germination in both HY and TNG67 mutants that is coincident with altered polyamine profiles. In particular, Put and Spd are increased in shoots of both cultivars, changes that are more pronounced in *put3* HY, while Spm is considerably enhanced in seed tissue of both *put3* HY and *put3* TNG67.

### Phenotypes of Os*put3* HY are abrogated by introduction of Os*PUT3*

**Figure 3:**
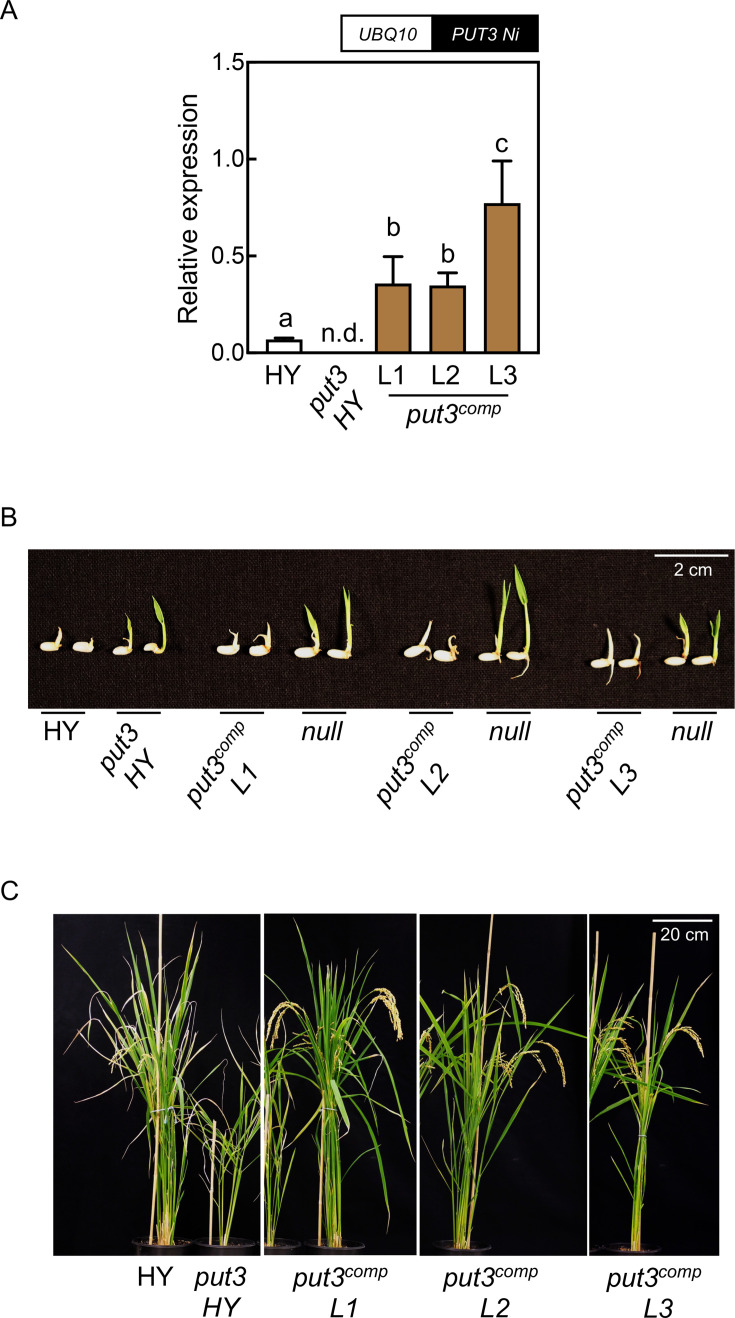
Complementation of rice *put3* HY. (**A**) A schematic representation of the transgene used for ectopic expression of *PUT3* from the Nipponbare cultivar (*PUT3 Ni*) expressed under the control of the Arabidopsis ubiquitin10 promoter is depicted at the top. Below is shown the RT-qPCR of *PUT3* expression of rice *put3* lines homozygous for the *PUT3* transgene (*put3^comp^*) in the T_3_ generation compared with the corresponding wild type HY cultivar and mutant. Plants were grown in ½ MS media under 12-hour illumination at 200 μmol photons m^-2^ s^-1^ at 28°C and 12-hour darkness at 20°C. Whole seedling tissue was harvested at ZT 6 from five-day-old seedlings. Transcripts were normalized to *UBQ5* (Os01g22490). Data are the mean ± SD of *N* = 3 or 4. Statistically significant differences (*P*<0.05) between means of genotypes were determined by one-way ANOVA with multiple comparisons and Dunnett’s test and are denoted by different letters. (**B**) Phenotypes of *put3^comp^* lines compared with the corresponding wildtype or null segregant grown in the presence of 1 μM paraquat. Plants were photographed five days after germination. The scale bar represents 2 cm. (**C**) Phenotype of mature plants as indicated grown under greenhouse conditions. The scale bar represents 20 cm.

**Figure 4:**
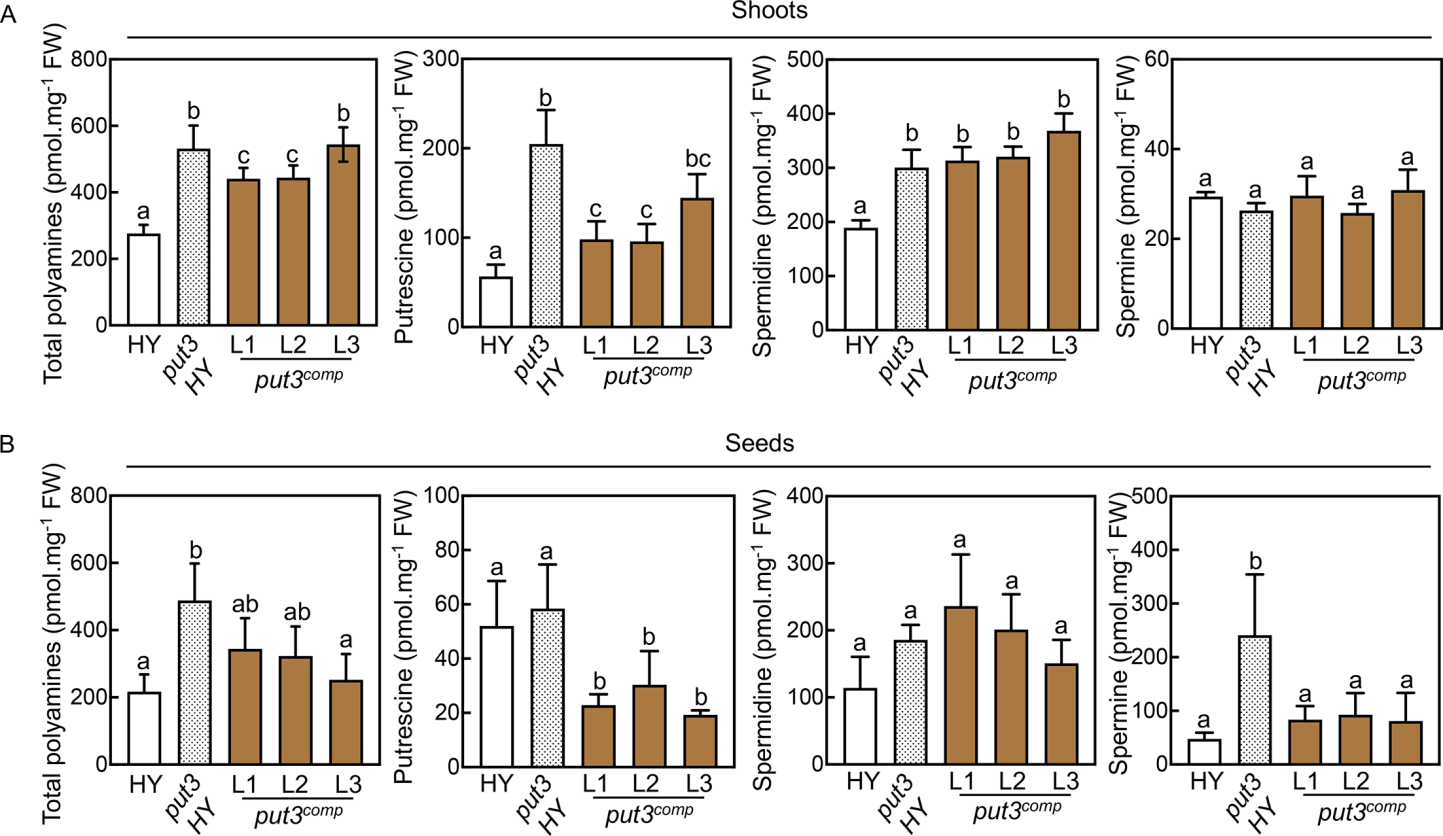
Polyamine content of complemented rice *put3* HY. (**A,B**) Total and individual polyamine levels for putrescine, spermidine, and spermine in lines as indicated either from shoots of 10-day-old seedlings (harvested at ZT 6) grown in ½ MS media under 12-hour illumination at 200 μmol photons m^-2^ s^-1^ at 28°C and 12-hour darkness at 20°C (**A**) or mature whole seeds of plants grown under greenhouse conditions (**B**). Data are the mean ± SD of *N* = 4. Statistically significant differences (*P*<0.05) between means of genotypes were determined by one-way ANOVA with multiple comparisons and Dunnett’s test and are denoted by different letters.

To validate the role of *PUT3* in the developmental impairments and PQ resistance observed in *put3* HY, we introduced a *PUT3* transgene from the Nipponbare cultivar (designated *AtUBQ10_pro_:PUT3* Nipponbare) previously shown to be associated with PQ resistance [6] by stable transformation. Three genetically independent lines were further characterized (assigned L1, L2, and L3), all of which had levels of *PUT3* expression statistically significantly higher than HY as determined by RT-qPCR and are referred to as *put3^comp^* (Figure 3A). In contrast with *put3* HY, the *put3^comp^* lines were sensitive to PQ, similar to HY (Figure 3B). Null segregants for the introduced transgene remained tolerant to PQ (Figure 3B). Moreover, the stunted growth and developmental phenotype of *put3* HY were abolished in these lines (Figure 3C). Furthermore, profiling the polyamine concentration of seedling shoots demonstrated that total polyamine levels were reduced in two of the lines. Notably, the levels of Put approached wild type levels in the three *put3^comp^* lines (Figure 4A). By contrast, Spd levels remained elevated and Spm levels were similar to wild type (Figure 4A). Measurement of the polyamine concentration of mature unpolished rice seeds indicated reduced accumulation of Spm in the *put3^comp^* lines compared with *put3* HY and was more similar to wild type levels (Figure 4B). Spd was not significantly changed among the lines, whereas Put was reduced in *put3^comp^* compared with either *put3* HY or wild type (Figure 4B).

## Discussion

A key aim in engineering PQ tolerance in crops is not penalizing agronomic traits such as yield or fitness. Preventing uptake and intracellular transport of PQ is an obvious strategy to achieve tolerance to this artificial herbicide [1]. Given that the *PUT* family encodes polycationic transporters that facilitate the transport of not only PQ but also polyamines and even thiamine (at least in *Arabidopsis*, although not the rice varieties studied here, but should be examined in other species), caution should be exercised in engineering PQ-tolerant varieties that target this gene family. Polyamines are heavily implicated in growth, development, and stress responses as outlined above, either directly in regulation of translation of key transcripts involved in signaling or indirectly through their catabolism triggered by stress that leads to the production of ROS [13,18,25]. Our study demonstrates that the down-regulation of *PUT3* expression confers tolerance to PQ in rice. Significantly, however, the disruption of *PUT3* has a considerable impact on the polyamine profile in the tissues examined.

In particular, the polyamine concentration of seeds is increased, most notably Spm. Interestingly, an increased level of Spd through a knockdown of Spm synthase (Spm is derived from Spd) was shown to increase germination rates in rice, suggesting that polyamine homeostasis is important during germination [26]. Indeed, the same study also showed that the ratio of Spd to Spm is critical for optimizing seed germination with low levels of Spm to Spd promoting germination. The increase in Spm in *put3* seeds thus disrupts polyamine homeostasis during germination and could account for the impairment in this process. Moreover, Spm has been shown to increase abscisic acid levels in *Medicago sativa* [27] and may do the same in rice, impairing germination as the hormone is a well-established inhibitor of germination [28]. The more pronounced increase in total polyamines in shoot tissue of *put3* HY compared with *put3* TNG67 presumably contributes to the severe stunting in seedling growth of the former. In contrast with seed tissue, it is Put and Spd that account for the increase in polyamines in shoot tissue of *put3* HY. However, the concentration of Put also increases in *put3* TNG67 and could imply that Spd is potentially more involved in the growth impairment in *put3* HY. Yet, Spd is not decreased in the *put3* HY complementing lines and thus increased Put is more likely to contribute to the morphological phenotype in this cultivar. The increase in Spm in root tissue also distinguishes *put3* HY from *put3* TNG67 and could also contribute to the growth defects in the former. Given the pleiotropic effects of polyamines and the need to maintain homeostasis, the precise cause of stunting of growth is difficult to decipher in *put3* HY. Notwithstanding, the transport of polyamine molecules is strongly linked with their biosynthesis and degradation and, in concert, achieve the tight regulation that maintains homeostasis [15]. This association may indicate different properties for the management of homeostasis between the two cultivars studied here, where transporter function in *put3* HY is more strongly associated with the maintenance of tissue polyamine levels. It is notable that germinated TNG67 seedlings share the morphological phenotype of wild type and had they not been examined for polyamine concentration could be considered ‘normal’. A recent study on CRISPR generated mutations of all three *PUT* genes in the japonica cultivar Xidao#1 reported no morphological differences to wild type under standard growth conditions in a growth chamber [19]. These lines were tolerant to concentrations of PQ higher (200 µM) than that used in the field (1.33–2.66 l of 300 g l^-1^ per sprayed hectare, approx. 140 µM), but careful examination of metabolic traits is warranted.

Even though both the HY and TNG67 *put3* mutants were tolerant to PQ, the divergent polyamine profiles of HY and TNG67 initially provided a hint that metabolic variance could be observed across rice cultivars. Indeed, studies indicate diverse polyamine profiles in natural rice cultivars at least in leaves [29,30], although the cultivars used here were not among those characterized. The disruption of *PUT3* and the corresponding alteration in polyamine concentrations clearly affect HY, while not having obvious morphological effects on TNG67, and reflect the relative importance of *PUT3* in different rice accessions. Even though fitness costs associated with herbicide tolerance are well documented and anticipated but not always observed [31,32], evolved PQ resistance in weeds is not reported to have a fitness cost but may not have been considered. Nonetheless, the association of PQ tolerance with polyamine transport and the association of polyamines with defense responses [13] may impart a trade-off in growth that is yet to be documented. Thus, in the case of crops, a large-scale screening of cultivars might reveal PQ tolerance without fitness costs, the basis of which may be a different resistance mechanism, or a different transporter that does not affect agronomic traits or key metabolites. The identification of polymorphisms may enable gene editing if appropriate for beneficial traits that lead to less PQ use in the field. Our study indicates that the improvement in germination of TNG67, with the right balance of polyamines, e.g., priming with Spd, would yield a rice cultivar that grows like wild type but is resistant to PQ.

The disruption of *PUT3* did not alter the thiamine profile of either the HY or the TNG67 cultivars in this study. This contrasts with *Arabidopsis*, where PUT3 is implicated in long-distance transport of thiamine and its mutation leads to lower amounts in tissues such as shoots and roots, negatively affecting growth [17]. *Arabidopsi*s *PUT3* expression is strong in phloem tissue and does not appear to be redundant in terms of thiamine transport with other *PUT* paralogs [17]; thus, species-specific differences in tissue expression and functional redundancy may rationalize variance between *Arabidopsis* and rice. The examination of 59 diverse rice varieties indicated up to four-fold differences in thiamine levels [24]. Among the varieties studied, TNG67 scored high for leaf thiamine concentration but was low in polished seeds. Here, our study shows that thiamine levels in HY are similar to TNG67 and are not changed in *put3* mutants, allowing us to conclude that the phenotypic differences are not a function of thiamine. However, other cultivars may behave differently with regard to phenotype due to diversity in metabolite traits.

In conclusion, the data presented here strongly implicate *PUT3* in the impaired development of *put3* HY that may be accounted for by altered polyamine levels. Elevated Put levels appear to correlate most closely with the shoot developmental defects. In addition, elevated Spm is associated with the loss of *PUT3* in seed tissue and could account for impaired germination. Therefore, despite *PUT* genes being perceived as good targets for engineering PQ resistance, their natural capacity to transport polyamines and thiamine should be assessed in detail because disabling functionality may negatively affect agronomic traits such as yield or stress resistance in the field.

## Material and methods

### Plant material and cultivation conditions

Rice wild type cultivars and the corresponding T-DNA insertion mutants at the *PUT3* locus (Os03g37984) were obtained from the Rice T-DNA Insertion Sequence Database in the HY background (*put3* HY, PFG_2B-60219.R) [22,33] and the Taiwan Rice Insertional Mutants collection [23] in the Tainung 67 (TNG67) background (*put3* TNG67*,* M0038724). For rice plant growth *in vitro*, wild type and *put3-*mutant seeds were dehusked and surface-sterilized in 70% (v/v) ethanol for 1 minute with shaking followed by gentle agitation for 30 minutes in 1.5% (v/v) bleach solution (Reactolab) containing 0.05% (v/v) Tween 20. Treated seeds were thoroughly rinsed in sterile water and air-dried. The sterilized seeds were sown on half-strength Murashige-Skoog basal salts media [34] (Duchefa) supplemented with 0.5 g l^-1^ 2-(*N*-morpholino) ethanesulfonic acid (MES (Acros), pH 7.0 adjusted with 10N NaOH, and 0.1 g l^-1^ myo-inositol (Sigma) and plant agar 3 g l^-1^ (Phytagel, Sigma) in sterile plastic tissue culture containers (Greiner, 330 ml volume containing 50 ml of medium). Seeds were stratified for two days in the dark at 28°C before being transferred to a climate chamber (Percival CU-22L) and grown under a 16-hour photoperiod at approximately 200 μmol photons m^−2^ s^−1^ generated by fluorescent lamps at 28°C and an 8-hour dark period at 20°C with 60–70% relative humidity and ambient CO_2_. PQ treatments (1 μM methyl viologen dichloride, Sigma) were carried out by inclusion of the chemical in the medium and growth under the same *in vitro* culture conditions. For growth of rice plants in soil, seeds were dehusked and germinated as above (without surface sterilization) on filter paper soaked with sterile water in a Petri dish, before transfer to soil under greenhouse conditions. Potting mix (Profi substrat classic, Einheits Erde, Germany) was treated by heating to 80°C for 40–60 minutes in a soil pasteurization unit (Sterilo, Harter Elektrotechnik, Germany) and irrigation with biological control agents Solbac (2.5 ml l^-1^, Andermatt Biocontrol, Switzerland) and Traunem (1 g l^-1^, Andermatt Biocontrol) prior to use. Soil mixtures for rice cultivation comprised three parts of treated potting mix, one part sand, one part perlite, and 800 g m^-3^ Osmocote fertilizer (Everis, Netherlands). Plants were grown in soil in 3-liter black plastic pots (19 cm diameter, Soparco, France) containing soil mixture prepared as above. Pots were placed on tables such that pots had water 3–5 cm above the level of the base of the pots. Water was changed twice per week, and plants were provided with Wuxal liquid fertilizer (Maag, Switzerland) at 0.2% (v/v), once at tillering stage and again at heading. A photoperiod of 12 hours of artificial lighting was provided year-round in the greenhouse, from MT400DL/BH EYE Clean Ace 400 W metal halide lamps (Iwasaki, Japan), supplemented by external ambient light conditions.

### Molecular characterization and binary vector construction for expression of Os*PUT3*

Genomic DNA from rice leaves was extracted according to Chen and Ronald [35] for genotyping and according to Sheu et al. [36] for Southern blot analysis. Total RNA was extracted from leaves using the NucleoSpin RNA Plant extraction kit (Macherey-Nagel) with ‘RAP’ (guanidinium-HCl) lysis buffer and 2-mercaptoethanol. An on-column DNaseI treatment was included during the procedure. Samples were eluted in sterile DEPC-treated water and stored at −80°C until use. RNA concentrations were quantified employing a Nanodrop ND-1000 (Witec) spectrophotometer. For cDNA synthesis, typically 1–3 μg was used and the Promega kit according to the manufacturers’ instructions. Briefly, RNA samples were treated with RQ1 DNase followed by addition of dNTPs, oligo(dT)_18_, Superscript II, and RNaseOUT for reverse transcription. RT PCR reactions were performed in 384-well plates using an Applied Biosystems AB7900 HT Fast instrument, using PowerUP SYBR Green Master mix (Applied Biosystems), forward and reverse primers (0.5 μM, see Supplementary Table S1), and one quarter volume of 10× diluted cDNA employing 40 cycles. The data were analyzed using the comparative cycle threshold method (2^−ΔΔCT^) [37] normalized to the reference genes *ACTIN1* (ACT1, Os05g36290) or *UBIQUITIN5* (*UBQ5*, Os01g22490) [38–40]. The coding region of *PUT3* from the Nipponbare cultivar was amplified from cDNA from 10-day-old seedlings using a proofreading polymerase and subcloned into the pENTR/D-TOPO vector that harbors *attL* recombination sites using the TOPO-TA cloning kit (Invitrogen) according to the manufacturers' instructions. The DNA sequence was subsequently cloned into Gateway compatible destination vector pUb-DEST vector harboring *attR* recombination sites for the expression driven by the Arabidopsis UBQ10 promoter [41], which carries the phosphinothricin N-acetyltransferase (*PAT*) gene for selection, annotated as *AtUBQ10_pro_:PUT3* Nipponbare.

### Genetic transformation of rice plants

The binary vector (*AtUBQ10_pro_:OsPUT3* Nipponbare) was introduced into *Agrobacterium tumefaciens* strain EHA105, and rice transformation, selection, and regeneration were conducted according to a previously described protocol [42]. Briefly, *put3* HY rice seeds were surface-sterilized (as above), and ten seeds were sown per plate (20 mm depth, Greiner Bio-One) on 50 ml of callus-induction media (‘N6D’). Plates were maintained for three to six weeks under continuous light (~80 μmol photons m^-2^ s^-1^) at 28°C until callus growth was abundant and creamy-colored after which cocultivation with the *Agrobacterium* strain followed by selection on phosphinothricin (25 mg l^-1^, Duchefa) was carried out. Shoot induction was initiated by transfer to MS-NK media containing phosphinothricin and continuous irradiance (~100 μmol photons m^-2^ s^-1^) at 29.5°C. Calli with shoot tissue 3–4 cm long were sectioned with a scalpel and transferred to MS-HF media in sterile plastic tissue-culture containers (Greiner, 330 ml volume containing 50 ml of medium). Regenerated plantlets showing robust root growth were transferred to soil and maintained under humid and partial shade conditions for four to five days to acclimatize, prior to transfer to greenhouse conditions to become established. T_1_ plants with apparent complementation of the *put3* HY dwarfism phenotype were carried forward. The presence of the transgene was confirmed by PCR (see Supplementary Table S1 for oligonucleotides used). Segregation for PQ sensitivity was also used to test for the presence of the transgene. Southern blot analysis was used to determine transgene copy number using a DIG-dUTP-labeled *PAT* probe synthesized using a PCR DIG probe synthesis kit (Roche).

### Determination of polyamine and thiamine content

Polyamines and thiamine compounds were extracted from homogenized, frozen plant material (∼25 mg). Polyamines were extracted using the method described in [17] typically using 10 volumes of 1% (v/v) trichloroacetic acid, hexamethylenediamine dihydrochloride as an internal standard and agitation for 30 minutes with glass beads (2 mm) followed by centrifugation at 16,100 ***g*** for 20 minutes. Polyamine determination was as described in [17]. Briefly, the supernatant was derivatized by adding two volumes of a saturated sodium carbonate (943 mM) solution, followed by four volumes of 75 mM dansyl chloride in 98% (v/v) acetone and incubation in darkness for 1 hour at 60°C, then supplemented with one volume of 2 mM L-proline and incubated for a further 30 minutes at room temperature to deactivate surplus dansyl chloride. Five volumes of ethyl acetate were then added, centrifuged at 16,100 ***g*** for 5 minutes, and the organic upper phase was vacuum concentrated at 45°C for 15 minutes. Samples were resuspended in methanol before analysis. Separation was performed using an Agilent 1200 series HPLC system on a C_18_ Ultrasphere 5 ODS column (250 mm × 4.6 mm, 5 μm particle size; Hichrom) under the following conditions: Solvent A = doubly distilled water; Solvent B = 100% methanol; 60–95% B in 23 minutes, 90–100% B in 2 minutes, 100% B for 5 minutes, returned to 60% B in 1 minute, and reequilibrated for an additional 9 minutes at a flow rate of 1.0 ml minute^−1^ and 27°C. Dansylated polyamines were identified by fluorescence (*λ*_ex_ = 365 nm; *λ*_em_ = 515 nm) and quantified by comparison with commercial standards [17]. Thiamine compounds were extracted as for polyamines. A 50 μl of supernatant was derivatized via the addition of 10 μl of 30 mM potassium ferricyanide and vortexed aggressively. Samples were incubated in the dark at room temperature for 10 minutes. Next, 15 μl of 1 M sodium hydroxide and 50 μl of methanol were added and mixed by vortexing. Samples were then centrifuged for 10 minutes at 16,100 ***g*** at room temperature, and the supernatant was used for analysis of thiamine compounds as described in [24]. Briefly, samples were chromatographed using an Agilent 1200 series HPLC system on a π-NAP column (150 × 4.6 mm, 3 μm particle size, Cosmosil. Two to fifty microliter volume samples were chromatographed using a methanol gradient under the following conditions: 0–20 minutes, 5–90% methanol; 20–21 minutes, 90–100% methanol; 21–25 minutes, 100% methanol; 25–26 minutes, 100–5% methanol; and 26–40 minutes, 5% methanol at a flow rate of 1 ml minute^-1^. Thiamine derivatives were identified by fluorescence, with excitation at 375 nm and emission at 450 nm. Peak area was used to calculate amounts of B_1_ vitamers against the standard curve from commercial standards based upon retention time. Data were normalized against tissue fresh weight or dry weight.

### Statistical analyses

GraphPad Prism version 10.2.0 was used for data analysis and statistical testing. Student’s *t*-tests, Wilcoxon tests, and one-way ANOVA with Dunnett’s *post hoc* test (all at *α* = 0.05) were used as indicated in the respective figure legends, wherein sample sizes are specified.

## Supplementary material

Online supplementary material

## Data Availability

The relevant data that support the conclusions of this study are contained within the article.

## Abbreviations

PQ: paraquat
PUT: polyamine uptake transporter
ROS: reactive oxygen species
