# Supplementary material for: Paraquat resistance mutations have differential effects on plant fitness in two rice cultivars
Source: Biochem J. 2025 Apr 23;482(8):401–12. doi: 10.1042/BCJ20240683 (PMC12203966; doi:10.1042/BCJ20240683)
Supplement: Online supplementary material [file BCJ-482-08-BCJ20240683-s001.pdf]

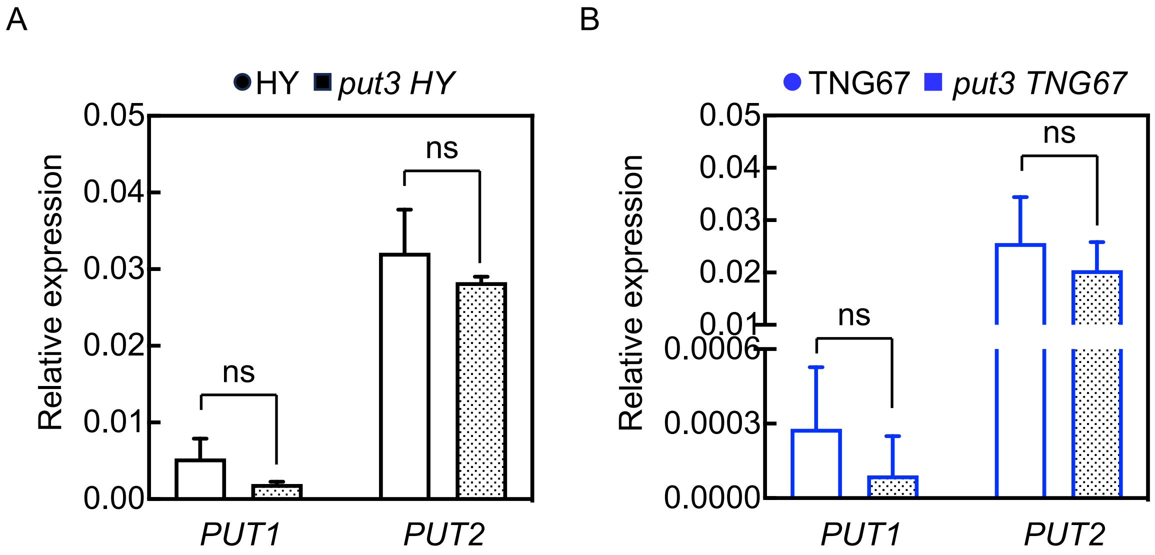

Supplemental Figure 1. *PUT1/2* expression in rice *put3* T-DNA insertion lines compared to the corresponding wild type cultivar. Plants were grown in ½ MS media under 16-hour illumination at 200 μmol photons m^{-2} s^{-1} at 28°C and 8-hour darkness at 20°C. Shoot tissue was harvested at ZT 6 from 12-day old seedlings. For (A) and (B), transcripts were normalized to *ACT1* (Os05g36290). Data are the mean ± SD of N=3. A *t*-test (α=0.05) was used to determine statistically significant differences between the means of expression of each gene in wild type and the corresponding mutant allele. Not significant (ns).

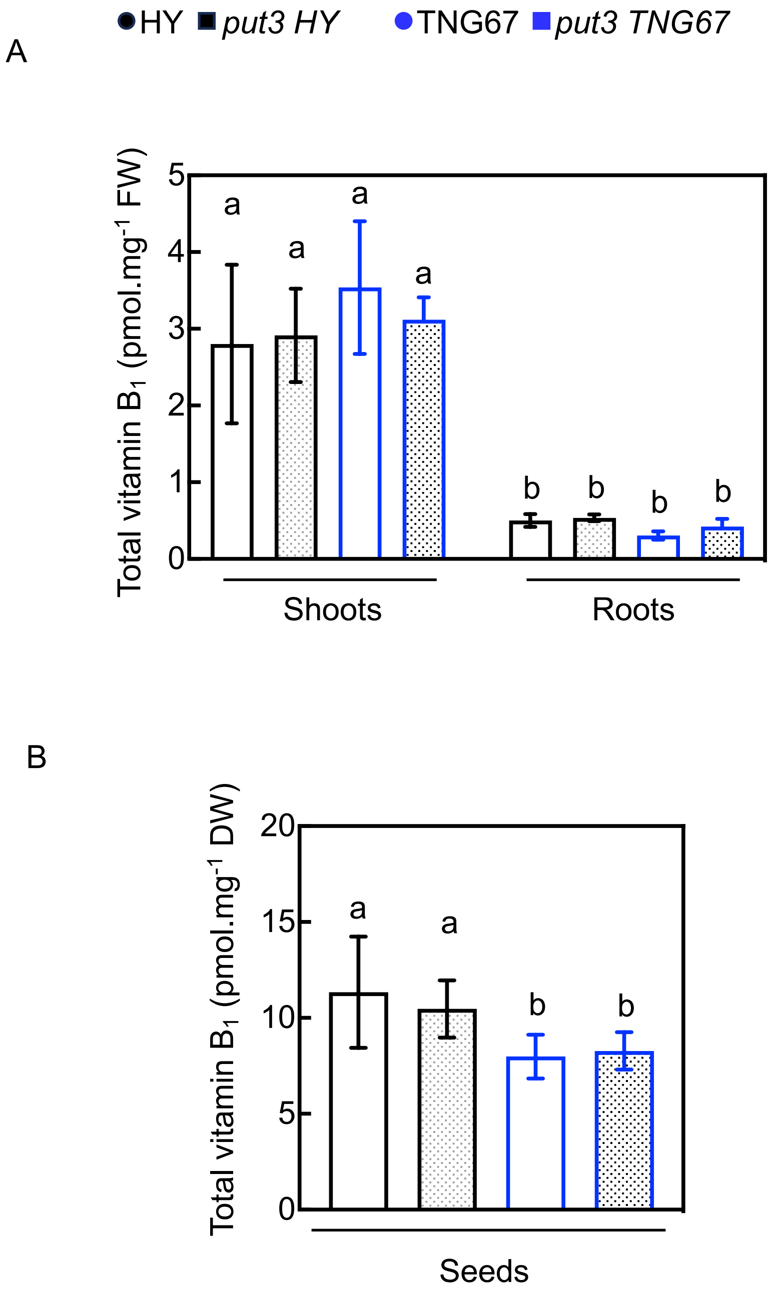

Supplemental Figure 2. Total vitamin B_1 content of rice *put3* T-DNA insertion lines compared to the wild type cultivar. (A) Total vitamin B_1 levels in shoots and roots of 10-day old seedlings grown in ½ MS media under 12-hour illumination at 200 µmol photons m^{-2} s^{-1} at 28°C and 12-hour darkness at 20°C. Tissues were harvested at ZT 6. (B) Total thiamine levels of mature whole seeds of plants grown under greenhouse conditions. Statistically significant differences ($P < 0.05$) between means of genotypes within each tissue type were determined by one-way ANOVA with multiple comparisons and Dunnett's test and are denoted by different letters. Data are the mean ± SD of N=3-5.

Supplemental Table 1. Nucleotide sequences of primers used in this study.

Name	Sequence (5′-3′)	Purpose
Os*ACTIN1*_F1 (Os05g36290)	ATCCTTGTATGCTAGCGGTCGA	RT-qPCR
Os*ACTIN1*_R1 (Os05g36290)	ATCCAACCGGAGGATAGCATG	RT-qPCR
Os*UBQ5*_F1 (Os01g22490)	ACCACTTCGACCGCCACTACT	RT-qPCR
Os*UBQ5*_R1 (Os01g22490)	ACGCCTAAGCCTGCTGGTT	RT-qPCR
Os*PUT3* (HY)_F1 Os03g37984	TCCAAAGAGAACACTCCCAAGG	RT-qPCR
Os*PUT3* (HY)_R1 Os03g37984	CAACCAGAAACCACCAAGAATTC	RT-qPCR
Os*PUT3* (TNG67)_F1 Os03g37984	TGGGCACTATGTTTCCTGAG	RT-qPCR
Os*PUT3* (TNG67)_R1 Os03g37984	ACACCACTCAGCCACTTTG	RT-qPCR
Os*PUT3* (TNG67)_F1 Os03g37984	CCGAAAATTAAGGAACACAGC	RT-qPCR
Os*PUT3* (Nipponbare)_F1 Os03g37984	CCGAAAATTAAGGAACACAGC	RT-qPCR
Os*PUT3* (Nipponbare)_R1 Os03g37984	GATGGAAACCTTTGGGACG	RT-qPCR
put3 (HY) WT_F1 Os03g37984	ATGGAGGATTGTGTTGGTATC	T-DNA genotyping
put3 (HY) WT_R1 Os03g37984	GCACACAAGTGGGATTGTGC	T-DNA genotyping
put3 (HY) T-DNA_F1 Os03g37984	ATGGAGGATTGTGTTGGTATC	T-DNA genotyping
put3 (HY) T-DNA_R1 Os03g37984	GGTGAATGGCATCGTTTGAA	T-DNA genotyping
put3 (TNG67) WT_F1 Os03g37984	ATCTAATCCCTGGGGTGTCC	T-DNA genotyping
put3 (TNG67) WT_R1 Os03g37984	TTCCCCAAGTCCATTTCAAGCC	T-DNA genotyping
put3 (TNG67) T-DNA_F1 Os03g37984	ACCAACGCTGATCAATTCCACAG	T-DNA genotyping
put3 (TNG67) T-DNA_R1 Os03g37984	TTCCCCAAGTCCATTTCAAGCC	T-DNA genotyping
OsPUT3 (Nipponbare)_F1 Os03g37984	CACCATGGAGGATTGTGTTGGTATC	Cloning
OsPUT3 (Nipponbare)_R1 Os03g37984	TCAGCACACAAGTGGGATTG	Cloning
